# Relationships between colored dissolved organic matter and dissolved organic carbon in different coastal gradients of the Baltic Sea

**DOI:** 10.1007/s13280-015-0658-4

**Published:** 2015-05-28

**Authors:** E. Therese Harvey, Susanne Kratzer, Agneta Andersson

**Affiliations:** Department of Ecology, Environment and Plant Sciences, Stockholm University, 106 91 Stockholm, Sweden; Department of Ecology and Environmental Science, Umeå University, 901 87 Umeå, Sweden

**Keywords:** CDOM, DOC, Baltic Sea, Secchi depth, Ecosystem

## Abstract

Due to high terrestrial runoff, the Baltic Sea is rich in dissolved organic carbon (DOC), the light-absorbing fraction of which is referred to as colored dissolved organic matter (CDOM). Inputs of DOC and CDOM are predicted to increase with climate change, affecting coastal ecosystems. We found that the relationships between DOC, CDOM, salinity, and Secchi depth all differed between the two coastal areas studied; the W Gulf of Bothnia with high terrestrial input and the NW Baltic Proper with relatively little terrestrial input. The CDOM:DOC ratio was higher in the Gulf of Bothnia, where CDOM had a greater influence on the Secchi depth, which is used as an indicator of eutrophication and hence important for Baltic Sea management. Based on the results of this study, we recommend regular CDOM measurements in monitoring programmes, to increase the value of concurrent Secchi depth measurements.

**Electronic supplementary material:**

The online version of this article (doi:10.1007/s13280-015-0658-4) contains supplementary material, which is available to authorized users.

## Introduction

Water quality in coastal areas is a result of processes in both terrestrial and marine ecosystems. Carbon is vital for living organisms and is therefore an important parameter to monitor. One of the main pools of carbon in coastal ecosystems is dissolved organic matter (DOM), which has both autochthonous and allochthonous sources. Allochthonous DOM consists mainly of degraded material of terrestrial origin, whereas autochthonous DOM is the result of biological activity in situ (Blough and Del Vecchio 2002; Gustafsson et al. 2014). Dissolved organic carbon (DOC) refers to the carbon in DOM. The light-absorbing colored or chromophoric fraction of DOM is commonly referred to as colored dissolved organic matter (CDOM).

The Baltic Sea is a semi-enclosed brackish sea with relatively high terrestrial input, restricted water exchange with the North Sea, and a slow turnover time of 20–30 years (Voipio 1981). About two-thirds of the DOC is allochthonous and the rest is autochthonous (Gustafsson et al. 2014) with a higher proportion of terrestrial DOC in the more northern basins (Deutsch et al. 2012). Furthermore, differences in catchment areas affect the DOC and CDOM pools (Skoog et al. 2011; Asmala et al. 2013). DOC and CDOM vary seasonally, with higher values in spring due to melt-water run-off from rivers (Ferrari and Dowell 1998; Stedmon et al. 2006) and higher photo-bleaching of CDOM in summer (Vodacek et al. 1997). Since most CDOM is river-derived, its concentration is inversely related to salinity, but the relationship differs among areas in the Baltic Sea (Kalle 1949; Jerlov 1976; Højerslev et al. 1996). The DOC entering lakes, estuarine, and coastal areas stimulates bacterial growth, which in turn affects other trophic levels in aquatic food webs (Hopkinson et al. 1998; Asmala et al. 2013). In the less productive northern part of the Baltic Sea, DOC entering the system is a crucial energy source for the ecosystem (Wikner and Andersson 2012; Asmala et al. 2013).

Colored dissolved organic matter absorbs light mainly in the ultraviolet and blue regions of the electromagnetic spectrum, and the absorption declines exponentially with increasing wavelength (Bricaud et al. 1981). Jerlov (1976) showed that the high CDOM absorption in the Baltic Sea has two effects: first, it reduces light transmission, making the Baltic Sea relatively dark, compared to other seas and oceans; second, it shifts the maximum transmission of light toward the red end of the spectrum, making the water brown, and reducing light available for photosynthesis. Colored dissolved organic matter influences the diffuse attenuation within the water column, and thus also the Secchi depth (Kratzer et al. 2003; Kowalczuk et al. 2006a; Nelson and Siegel 2013). High CDOM absorption has a negative effect on primary production by limiting the light penetration, thus reducing the euphotic zone depth and the quantity and spectral quality of the photosynthetic active radiation (PAR) available for photosynthesis (Ask et al. 2009; Urtizberea et al. 2013).

As chemical analysis of DOC is costly and time consuming, while CDOM absorption measurements are relatively cost-efficient and quick, the latter may be an alternative means of quantifying dissolved organic carbon (Asmala et al. 2013; Nelson and Siegel 2013). However, as the relationships between CDOM content and DOC depends on many factors, it is important to investigate local relationships. Despite its ecological importance in aquatic ecosystems, the role and the inter-relationships of CDOM in the Baltic Sea basins are relatively poorly studied, especially for coastal areas. Most studies are from the southern and western Baltic Sea and only a few are comparative (e.g., Schwarz et al. 2002; Skoog et al. 2011).

Colored dissolved organic matter absorption can be estimated by remote sensing, and is one of the main products of ocean color remote sensing data. However, as the relationship between CDOM and DOC varies both regionally and seasonally, global algorithms are unlikely to work, and regional ones need to be developed (Kowalczuk et al. 2010b; Matsuoka et al. 2013; Mannino et al. 2014). Some Baltic Sea monitoring programs provide long time-series of DOC and CDOM fluorescence, but no CDOM absorption at 440 nm. In order to improve coastal monitoring, we therefore need to derive the relationships between CDOM absorption (g440) and DOC, and between CDOM absorption and CDOM fluorescence. This way one can much better combine conventional ship monitoring with ocean color remote sensing. In recent times, remote sensing of chlorophyll has become the main tool for estimating global primary production and is thus the basis for most models of marine food-webs and ecosystems. While remote sensing algorithms already work well for the open ocean, it is vital to further improve our understanding of optically complex coastal waters. Absorption in the blue by terrestrial CDOM interferes with remote sensing chlorophyll algorithms, especially in areas with high CDOM concentrations, like the Baltic Sea. If the high CDOM absorption is not taken into account, chlorophyll and hence primary production can be over-estimated. The potential fisheries yield calculated from estimated primary production in models can then also be over-estimated, as in Conti and Scardi (2010) where primary production in the Baltic Sea was estimated to 10 times its real value. Hence, in situ data are necessary to develop reliable remote sensing algorithms for high CDOM waters.

Another important aspect is the influence of climate change. In projected climate change scenarios for boreal regions, the loads of organic matter (DOM/DOC and CDOM) transported into surface waters increase due to climate warming and increased precipitation, leading to higher terrestrial input as well as lower salinity in northern Baltic Sea (Meier 2006; Larsen et al. 2011; Meier et al. 2012a). This has implications for the monitoring and adaptive management of Baltic Sea eutrophication, as higher CDOM loads most likely will affect light attenuation and also Secchi depth, which is commonly used as an indicator of eutrophication. This is likely to be important not only in the Baltic Sea, but also for all coastal waters with considerable terrestrial loading of DOM.

This paper studies the relationships between CDOM, DOC, salinity, and Secchi depth in coastal gradients in the Gulf of Bothnia (GB) and the Baltic Proper (BP). The GB has high terrestrial input and low salinity whereas BP generally has lower terrestrial input and somewhat higher salinity. We expect higher concentrations of both DOC and CDOM in the GB, especially in spring, when greater freshwater inflow transports more organic material into the coastal waters. Furthermore, we expect the ratio between CDOM and DOC to be higher in the GB than in the BP due to the higher run-off of humic substances. We hypothesize that the origin and amount of terrestrial run-off in the different coastal areas will be reflected in the relationships between CDOM and DOC, CDOM and salinity, as well as the CDOM absorption and CDOM slope coefficient. The relationship between CDOM and Secchi depth was also studied.

## Materials and methods

### Sampling sites

**Fig. 1:**
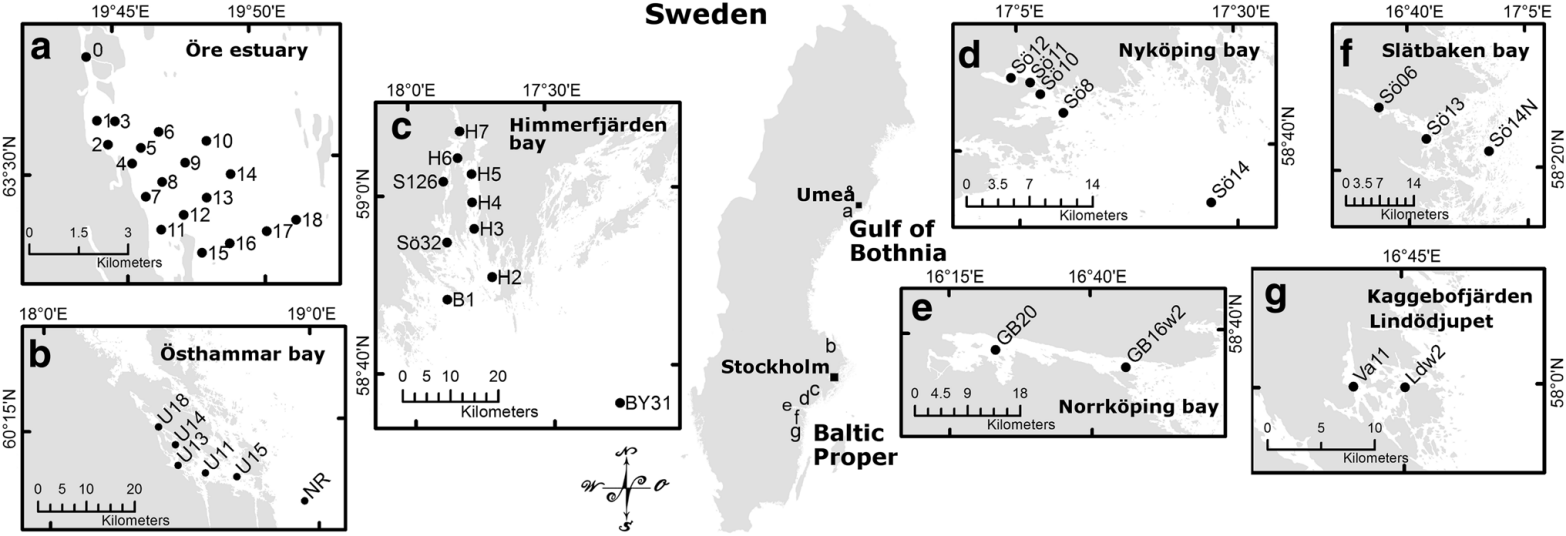
Map of the studied coastal areas (gradients **a–g**) and locations within the Baltic Sea, along the Swedish east coast. Map © Lantmäteriet, Gävle 2014, permission I 2014/00691

Water quality parameters were measured in Swedish Baltic Sea coastal waters of the Western Gulf of Bothnia (GB) and the North Western Baltic Proper (BP) (Fig. 1). In the GB, we sampled a gradient in the Öre estuary, a relatively open archipelago where freshwater from the Öre River creates a salinity gradient. In the BP, six coastal areas were investigated from north to south: Östhammar bay is a shallow area with no major freshwater inputs and hence a weak salinity gradient. It receives the effluent of an Urban Waste Water Treatment Plant (UWWTP); so does Himmerfjärden, a large, elongated bay divided by several sills, with low freshwater input and a weak salinity gradient. The Nyköping gradient (NG) is a shallow bay, with a large freshwater input from three rivers, and restricted water exchange in the inner localities. The city of Norrköping is located at the head of Bråviken bay, which is a mostly shallow 50-km-long bay with a large fresh water inflow. Slätbaken bay is rather deep with sills restricting the water exchange, heavily eutrophicated and has a freshwater input with a salinity gradient. Two other locations, south of Slätbaken bay, were also included.

### Field sampling

Surface water samples were collected in 4 years (2010–2013) using standard sampling methods (e.g., Niskin or Ruttner bottles, buckets). Samples were stored cold in plastic or amber glass bottles for a maximum of 24 h, but most often filtered directly after sampling. A total of 171 stations from 19 locations were sampled in the GB from May to early September 2010 (18 sea locations and one location in Öre River). The 143 stations from 27 locations in the BP were mostly sampled in June, July, and August 2010–2013, with additional spring data from Himmerfjärden in 2010 and 2012.

### Optical and chemical analyses

After filtration through a 0.22-μm membrane filter, the CDOM absorbance of the water sample was measured between 300 and 800 nm in a 10-cm quartz cuvette in a dual-beam Shimadzu UVPC 2401 spectrophotometer. A baseline was set with Milli Q water in both beams, and each sample was measured against Milli Q water as a blank (Kratzer 2000; Kratzer and Tett 2009). The absorbance was corrected for scattering by subtracting the average absorbance between 700 and 750 nm. To derive the CDOM absorption coefficient, g440, the absorbance was multiplied with 2.303 and divided by the length of the cuvette (Kirk 2011). The absorption at 440 nm was used because it corresponds approximately to the second photosynthetic peak of chlorophyll absorption and is one of the standard products of remote sensing data. The exponential slope coefficient, *S*, was calculated from the least square linear regression fit of the log values of the absorption curve between 350 and 500 nm (Blough and Del Vecchio 2002; Kirk 2011). The relative standard error for the CDOM method was calculated and gave a precision of 6 % for g440 and 4 % for *S*. In the GB, CDOM was also measured using fluorescence spectroscopy, CDOM_fl_ (analyzed according to HELCOM 2014 with 350/450 nm ex/em). The DOC concentrations were analyzed according to Swedish Standards (SIS 1997). For all locations, except the freshwater Öre River station, salinity (Practical Salinity Scale, PSS-78 or conductivity meter, SevenEasy S30) and Secchi depth were measured. Accredited chemical laboratories at Umeå and Stockholm Universities analyzed DOC, salinity, and CDOM_fl_.

### Statistics

Generalized linear models (GLMs) were used to test if the intercepts and slopes differed among areas for the relationships of g440 to DOC/salinity and Secchi depth as well as for DOC versus salinity. Generalized linear models were also used to investigate the relationship between g440 and DOC versus CDOM_fl_. For all linear GLMs, a Gaussian distribution family was used. The non-linear GLMs for CDOM absorption and Secchi depth were fitted with a Poisson distribution family and a log link. Non-parametric Kruskal–Wallis tests or ANOVA were used for seasonal analyses in g440, *S* and for the CDOM:DOC ratio. The CDOM absorption slopes are negative, but are here presented as absolute values, with higher values indicating steeper slopes. For all statistical analyses assumptions of independence, normality and heteroscedasticity were tested and residuals analyzed. For the empirical models, the root mean square errors (RMSE) and normalized RMSE (i.e., RMSE divided by the range of *y*-observations) were assessed. The confidence level used was 0.05 and the number of observations (*n*) is given for each analysis. R 3.0.1 was used for statistical and data analyses as well as graphs (R Core Team 2013).

## Results

**Fig. 2:**
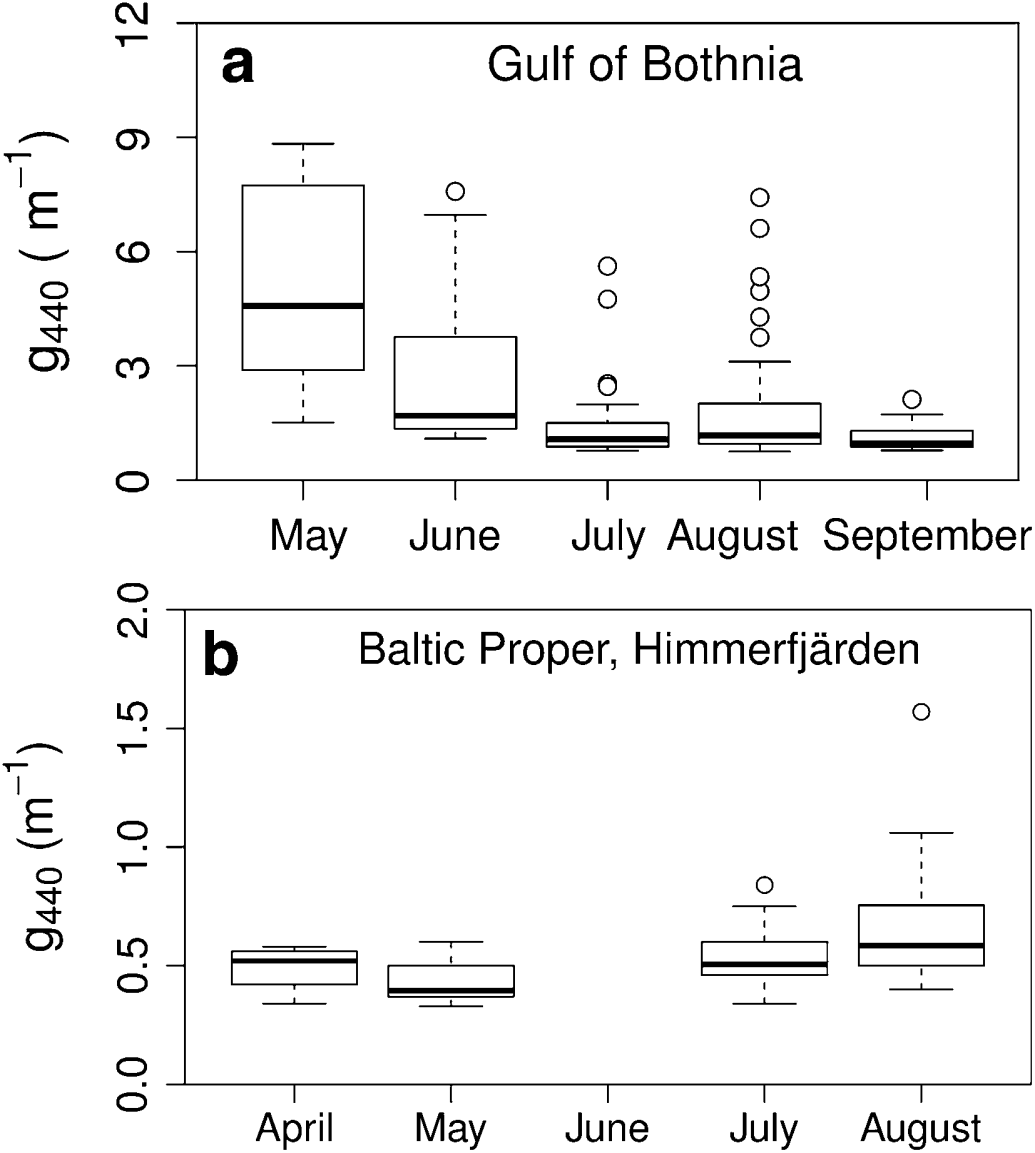
Seasonal analysis of CDOM absorption (g440, m^−1^) per month. Graph **a** shows the results from the Gulf of Bothnia and **b** from the Baltic Proper, exemplified by the Himmerfjärden bay (no data in June). *Horizontal lines* in the boxplots indicate the median values, *horizontal edges* of the boxes indicate the 25th and 75th percentiles, the *whiskers* indicate the minimum and maximum observations within 10th and 90th percentiles, *open circles* represent outliers

The CDOM absorption was higher and more variable in the GB than in the BP (Fig. 2; Fig. S1; Table S1; S denotes Supplementary Material). The DOC concentrations were in the same range for both areas, with the highest DOC concentrations in the BP at the inner stations of the NG (Table S1). The mean Secchi depth was larger in the BP than in the GB (Table S1). In general, g440 decreased with increasing salinity, associated with distance from land.

In the GB, g440 was significantly higher in May and June than in July, August and September (Kruskal–Wallis test; *p* < 0.001, df = 4, *χ*^2^ = 67.4, *n* = 169). Values were lower in June than in May, indicating lower terrestrial input, but still significantly higher than in July (Fig. 2a). The outliers in the g440 data in August (Fig. 2a) were due to a second peak in river outflow. The differences in CDOM slopes among months were tested with ANOVA. May and June showed the same patterns as for g440; differing from each other and from July, August, and September (*p* < 0.001, df = 4, *n* = 169) (Fig. 2b).

**Fig. 3:**
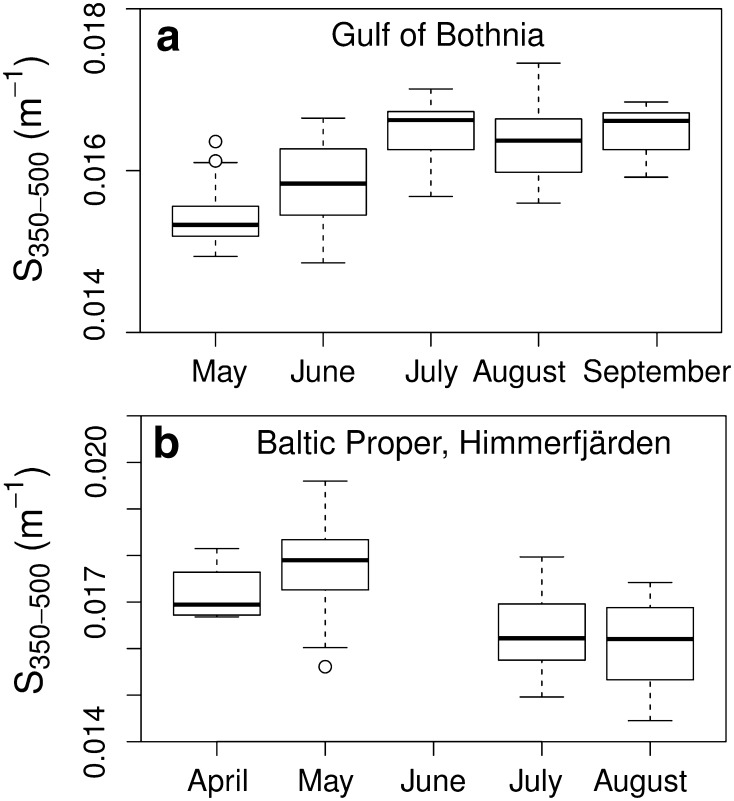
Seasonal analysis of CDOM slope (*S*, m^−1^) per month. Graph **a** shows the results from the Gulf of Bothnia and **b** from the Baltic Proper, exemplified by the Himmerfjärden bay (no data in June). Boxplots are as in Fig. 2

In contrast, g440 in Himmerfjärden bay in the BP was slightly higher in summer than in spring. The only significant difference was between May and August (Kruskal–Wallis test; *p* < 0.001, df = 3, *χ*^2^ = 21.4, *n* = 64) (Fig. 3a). No data were available from June. In Himmerfjärden, CDOM slopes were significantly steeper in May than in July and August (ANOVA, *p* < 0.001, df = 3, *n* = 64) (Fig. 3b).

### Empirical relationships

**Fig. 4:**
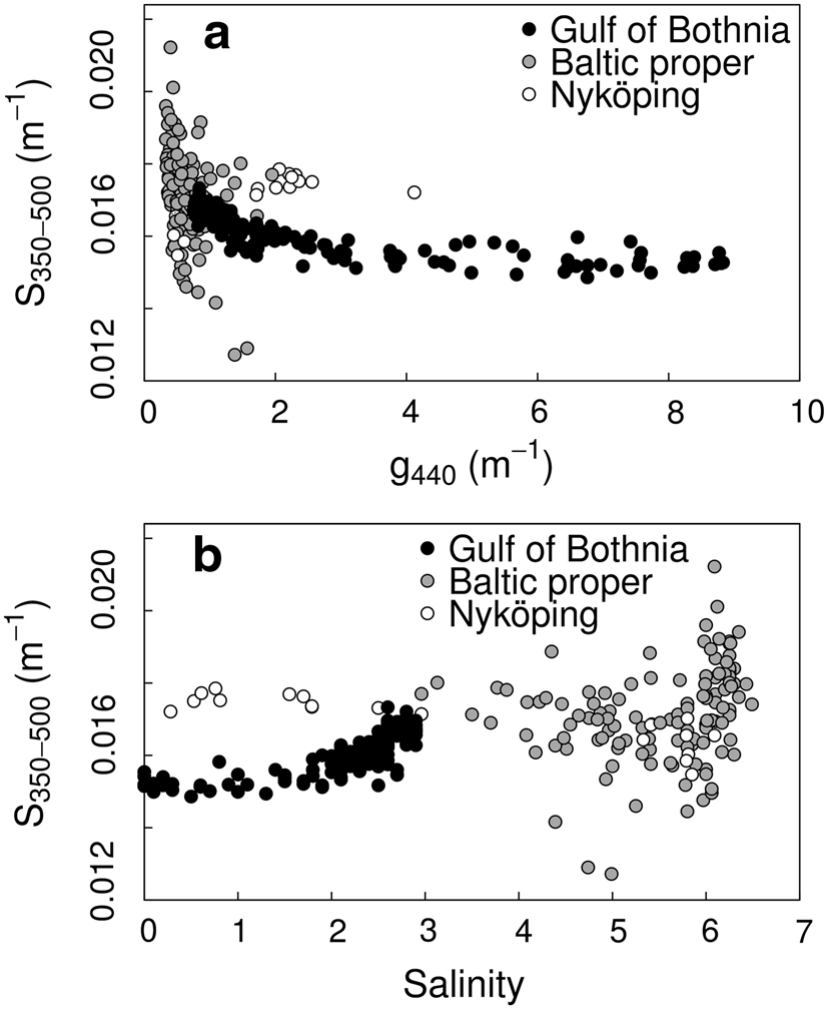
Relationship between CDOM slope coefficient (*S*, m^−1^) and **a** CDOM absorption (g440 m^−1^), and **b** Salinity. *Black dots* represent data from the GB and *gray dots* are pooled data from the BP, *open circles* are data from the Nyköping gradient in the BP

The variability of the CDOM slope, *S*, was higher in the BP than in the GB, especially at lower g440 values (Fig. 4a) and with higher salinities (Fig. 4b). Although the *S* values had a narrower range in the GB, the variability in g440 was very high (Fig. 4a; Table S1). In the BP, no general relationships could be seen, as *S* varied greatly over the same range of g440 and salinity (Fig. 4; Table S1). The Nyköping gradient (NG) was an exception, with relationships that differed clearly from the rest of the BP, as well from the GB.

**Fig. 5:**
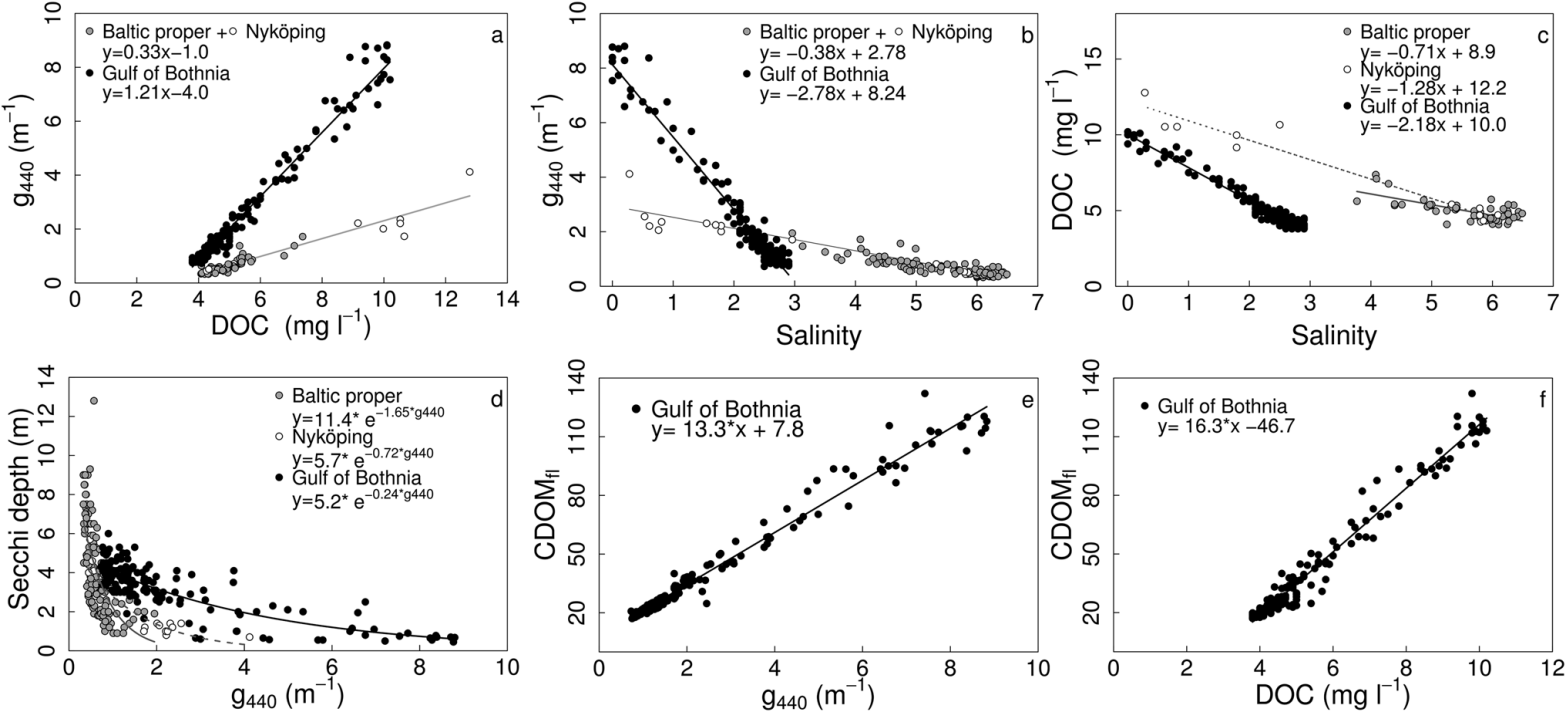
Relationships between g440 and DOC (**a**), g440 and Salinity (**b**), DOC and salinity (**c**), and Secchi depth and g440 (**d**) for different areas. The relationships for CDOM absorption measured by fluorescence over g440 (**e**) and DOC (**f**) in the Gulf of Bothnia. The *black dots* and *black lines* are data from the GB, the *gray dots* and *gray lines* denote pooled data from the BP, and *open circles* and *dashed lines* are data from the Nyköping gradient in the BP

Both the slope and the intercept of the relationship between DOC and g440 differed significantly between GB and BP (Fig. 5a; Table 1). The linear model had a steeper slope in the GB, resulting in much higher g440 absorption per DOC concentration. For example, a value of 10 mg l^−1^ DOC would relate to a g440 absorption value of 2.3 m^−1^ in the BP and 7.9 m^−1^ in the GB. This gives up to 3.5-fold higher CDOM absorption values in the GB than in the BP. The relationship between g440 and salinity differed significantly in both slopes and intercepts between the two study areas (Fig. 5b; Table 1). In the GB, g440 values were higher at a given salinity than in the BP, and the slope was much steeper (−2.8 vs. −0.38). Therefore, basin-specific g440 and salinity dependences can be identified. The higher g440 at lower salinities at the inner stations in the NG followed the same relationship as the other BP data.

**Table 1:** Results and equations from the relationships between g440 vs. DOC, g440 vs. salinity, DOC vs. salinity, Secchi depth (SD) vs. g440, and CDOM_fl_ vs. g440 and DOC tested with GLMs on data presented in Fig. 5. Error statistics for each equation: root mean square error (RMSE) and normalized root mean square error (NRMSE)

Area	Equation	*R* ^2^	*p* value	*n*	RMSE	NRMSE (%)	GLM model	*χ* ^2^	df_factor_	df_resid_	*p* value	
											Slope	Intercept
GB	g440 = 1.2 × DOC − 4.0	0.97	<0.001	170	±0.46	6	g440~DOC × area	119	1	233	<0.001	<0.001
BP	g440 = 0.33 × DOC − 1.0	0.89	<0.001	67	±0.20	5						
GB	g440 = −2.7 × salinity + 8.20	0.95	<0.001	162	±0.48	6	g440~salinity × area	358	1	300	<0.001	<0.001
BP	g440 = −0.38 × salinity + 2.80	0.87	<0.001	143	±0.24	6						
GB	DOC = −2.18 × salinity + 10.0	0.96	<0.001	161	±0.33	5	DOC~salinity × area	57	2	223	<0.001	<0.001
BP	DOC = −0.71 × salinity + 8.90	0.5	<0.001	53	±0.49	15						
NG	DOC = −1.28 × salinity + 12.2	0.95	<0.001	12	±0.68	8						
GB	SD = 5.2 × e^−0.24 × g440^	0.66	<0.001	161	±0.43	8	SD~g440 × area	56	2	296	<0.001	<0.005
BP	SD = 11.4 × e^−1.64 × g440^	0.42	<0.001	117	±0.75	9						
NG	SD = 5.7 × e^−0.72^ ^× g440^	0.42	<0.001	20	±0.49	14						
GB	CDOM_fl_ = 13.3 × *x* + 7.8	0.98	<0.001	169	±4.7	4	CDOM_fl_~g440				<0.001	<0.001
GB	CDOM_fl_ = 16.29 × DOC − 46.67	0.97	<0.001	170	±5.1	4	CDOM_fl_~DOC				<0.001	<0.001

Based on statistical results, the relationship between DOC and salinity was evaluated separately for the three areas GB, BP and NG. Hence, the GLM was fitted with three factors for the predicting parameter; area. The relationships differed for all three areas (Fig. 5c), with both slopes and intercepts significantly different (Table 1).

To study the connection of CDOM absorption to Secchi depth, a GLM was fitted with Secchi depth as a response to g440. The results showed that all three areas had different exponential relationships, with different slopes and intercepts (Fig. 5d; Table 1). In the GB the slope was not very steep as Secchi depth decreased almost linearly with higher g440. The Secchi depth in the BP and in the NG showed a clearer exponential decrease with increasing g440. The Secchi depth varied greatly at low g440 values, both for the BP and NG.

To link different monitoring methods the CDOM_fl_ was plotted against g440 and DOC (GB only). The CDOM_fl_ showed strong, significant positive relationships both to g440, and to the DOC concentration (Fig. 5e, f; Table 1).

**Fig. 6:**
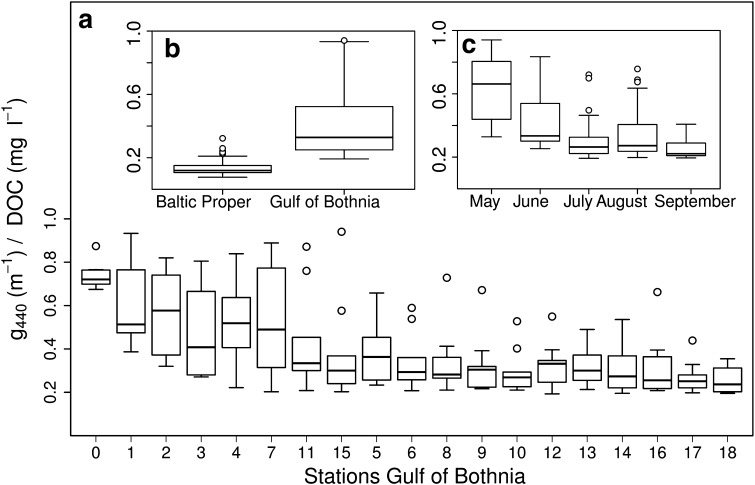
Box plots showing the CDOM:DOC ratios (l m^−1^ mg^−1^) for **a** spatial variations in the GB, **b** areas, and **c** monthly variations in GB (the latter two inserted). The stations are roughly placed along the decreasing influence from the freshwater input from the Öre River with the Öre River station (0) to the left and the furthest open sea station (18) to the right. Boxplots are as in Fig. 2

To further compare the relationship between CDOM absorbance and DOC for different areas and months, the CDOM:DOC ratio was calculated. In the GB, the ratios decreased from the stations closest to the river outflow to the open sea (Fig. 6a). The variability was quite high for all locations, except for the Öre River station and the open sea locations. The highest variability was seen at the stations where the mixing between freshwater and seawater occurs. The areas were statistically different (Kruskal–Wallis tests, *p* < 0.001, df = 1, *χ*^2^ = 131.2, *n* = 238), with higher ratios (closer to 1) in the GB than in the BP (Fig. 6b). There were no differences between the gradients within the BP. Furthermore, there were significant seasonal differences in the GB, with higher ratios in May but also a difference between June and September (Kruskal–Wallis test; *p* < 0.001, df = 4, *χ*^2^ = 67.1, *n* = 170) (Fig. 6c).

## Discussion

Based on the empirical relationships we found the two main water bodies studied, the Öre estuary in the GB and the coastal waters of the BP (except the inner stations in NG), to have clearly different CDOM pools and optical properties. This was consistent for several of the investigated relationships, such as between *S* and g440; *S* and salinity; DOC and salinity as well as g440 and Secchi depth. The CDOM:DOC ratio differed between areas, as well as with season in the GB and spatially in the Öre estuary.

The seasonal variation in the GB, with significantly higher terrestrial input in spring, and higher g440 values and steeper slopes (higher *S*) (Fig. 2) means that the CDOM pool entering the system during spring is different, presumably affected by snow melt. The high variability in August in GB was due to renewed high freshwater inflow after heavy rains. The optical signals changed with season also in the BP but, unexpectedly, in the opposite direction. This may indicate greater autochthonous CDOM production during the summer bloom in the BP. Seasonal changes in *S* and CDOM absorption have been observed previously, in the southern Baltic Sea, between summer and autumn for CDOM absorption, but with no statistical difference between spring and summer (Kowalczuk et al. 2005). In the Middle Atlantic Bight, *S* values were lower in March than in April, but no seasonal change in CDOM absorption could be seen (Vodacek et al. 1997).

### Optically different water masses

The empirical relationships between *S* and g440, and *S* and salinity, suggest that we deal with three different water masses. In the GB, the *S* values at the lowest salinities and the highest g440 values are more influenced by freshwater input, and DOC contains more allochthonous material, whereas older and more broken down autochthonous material is associated with lower g440 and higher salinities. The trend for *S* indicates that the CDOM pool at the open sea locations in the GB is composed of older, more broken-down material (steeper slopes), affected by bacterial degradation and photobleaching (Vodacek et al. 1997). The relative slope values found in this study agree with the results of Carder et al. (1989), with more humic acids in the GB generating lower slopes whereas more fulvic acids (i.e., smaller and more broken-down molecules) associated with higher slope values were found in the BP. In the BP (with lower g440 values and high salinity) *S* varied more than in the GB. This high variability may be associated with autochthonous CDOM production by algae, and CDOM loss by bacterial breakdown (Carder et al. 1989) and photobleaching (Vodacek et al. 1997; Stedmon et al. 2000). However, it could also be due to mixing of an older, background CDOM pool, with newer terrestrial and marine material. Kratzer and Tett (2009) found that only 8 % of the CDOM variance could be explained by chlorophyll in the Himmerfjärden bay. The CDOM pool at the inner locations of the NG seems to consist of newer material (higher g440 and steeper slopes), different from the open sea locations of both the BP and the GB. A difference in the relationships between *S* and g440 and salinity caused by differences in the CDOM pool was seen between fjord water (with lower salinity) and open sea water in Denmark (Stedmon et al. 2000). A linear relationship between *S* and log (g400) with a decrease in *S* with higher CDOM was also found by Kowalczuk et al. (2005) in the Southern Baltic Sea. Visually the pattern for *S* dependencies on CDOM absorption (Fig. 4a) are very similar to Vodacek et al. (1997) and Kowalczuk et al. (2006b). In Kowalczuk et al. (2006b), the pattern is explained by a conservative mixing model between high g440 values with low *S* and low g440 values with higher *S*, as a proxy for riverine and marine CDOM pools. However, as there are no very low g440 values in GB related to higher *S* and vice versa for BP, the conservative mixing model can only be applicable to the GB data and in the NG gradient, but not generally in the BP. These results confirm that the BP is less influenced by terrestrial material than the GB. Still, the Baltic Sea is generally more affected by allochthonous DOM than other seas (Højerslev et al. 1996; Schwarz et al. 2002). Although the *S* values have been calculated differently in different studies, the relative difference should still be the same (Blough and Del Vecchio 2002).

### CDOM and DOC relationships

Strong relationships between CDOM and DOC similar to ours, have been found in other Baltic Sea areas (Vodacek et al. 1997; Stedmon et al. 2000; Kowalczuk et al. 2010a). The relationship between g440 and salinity are also in line with other studies (Kowalczuk et al. 2006b; Skoog et al. 2011). Differences in the CDOM to salinity relationship between Baltic Sea areas have been described before by Højerslev et al. (1996), but our results with the NG coastal data, show that the BP relationships hold for even lower salinities. The three areas showed distinctive relationships between DOC and salinity, with a strong freshwater influence in the NG. A possible explanation could be the difference in land use, with more agriculture in the drainage area of the BP, and especially the NG (Sweitzer et al. 1996). Hence, the BP and the NG may show stronger anthropogenic influence and the GB more natural influence by bogs, wetlands and forests. The different relationships of g440 and DOC to salinity show that CDOM and DOC are not strongly coupled in the Baltic Sea, with different relationships between the areas. However, CDOM has a more general relationship to salinity than DOC within the BP, as the relationship was consistent for CDOM but not for DOC.

The study by Skoog et al. (2011) showed a partial decoupling between TOC and humic fluorescence within the Baltic Sea, as the humic fluorescence decreased from north to south, whereas the TOC changed little. We found higher DOC concentrations in local coastal areas of the GB and in the NG. In the BP and some of the GB locations, less influenced by freshwater, DOC concentrations were approximately the same, ~4–5 mg l^−1^. Our results confirm the partial decoupling, as the CDOM:DOC ratios were higher in the GB than in the BP. Furthermore, the seasonal differences in the GB showed that CDOM and DOC are more strongly linked in spring (higher ratios), when more new terrestrial material enters the system. This means that CDOM cannot be used as a general proxy for DOC, as confirmed by different relationships among the areas.

### Monitoring of CDOM and DOC by remote sensing

As DOC is regularly measured in the Swedish marine monitoring programs, the relationships reported here could be used to approximate CDOM from DOC for the respective areas, with the uncertainty estimated by the regression analysis. The need for locally adapted models and well evaluated data input to biogeochemical or productivity models is clear. Colored dissolved organic matter is, however, measured within HELCOM as CDOM fluorescence and the results from this study are in line with the relationships found by Vodacek et al. (1997) and Kowalczuk et al. (2010a). Our results show that g440 can be estimated from fluorescence monitoring measurements also in the GB (Fig. 5e). Colored dissolved organic matter can be derived from remote sensing, making it possible in the future to incorporate such measurements in regular monitoring programs. However, further understanding of the optical properties of the CDOM dynamics is required for improving remote sensing algorithms, especially in regions with high CDOM concentrations. Kratzer (2000) and Schwarz et al. (2002) showed that the Baltic Sea has a very different optical signal from other seas. Valuable studies have been carried out to develop remote sensing algorithms for deriving CDOM and DOC (Kowalczuk et al. 2010b; Matsuoka et al. 2013; Mannino et al. 2014). The in situ data ranges and optical relationships presented in this study can be used to train a region-specific processor for the improved retrieval of CDOM absorption, but the results also highlight the challenges for remote sensing in the Baltic Sea. The high variability of *S* demonstrates the difficulty in developing general algorithms for CDOM retrieval from remote sensing data. Moreover, remote sensing uses the reflectance at 440 nm to derive both CDOM and chlorophyll-*a*, but the considerable high CDOM absorption makes the signal very weak in the blue, with high errors. Therefore, retrieving chlorophyll-*a* values is also a challenge in the Baltic Sea, especially in the GB, due to the low reflectance caused by the high CDOM absorption (Berthon and Zibordi 2010). Knowledge on the complex optical properties are required for the development of other remote sensing algorithms as well, for example, chlorophyll-*a*, diffuse attenuation coefficient of down welling irradiance, *K*_d_, total suspended material, *K*_d_ (PAR) and also Secchi depth.

### CDOM climate change and Secchi depth

Climatological models project that the river runoff will increase from the Baltic Sea catchment, leading to lower salinity but increased input of DOM, including CDOM (Meier et al. 2012a, b). These scenarios assume increased “brownification” of natural waters in northern Europe. Our results indicate that if more CDOM enters the sea, it will negatively affect the under-water light climate, with less light penetrating the water (Secchi depth decrease), reducing the energy reaching the phytoplankton and lowering primary production (Ask et al. 2009; Lefébure et al. 2013).

From a management point of view, Secchi depth is an important parameter, because it is the water quality indicator with the longest measurement record in the Baltic Sea (Meier et al. 2012b). However, studies of the relationship between CDOM and Secchi depth are scarce in Sweden. The large difference in this relationship among the areas in this study was unexpected. The fresher terrestrial material associated with higher CDOM in the GB seems to have a strong influence on the Secchi depth. The high variability of the Secchi depth in coastal areas with low g440 in the BP indicates that the Secchi depth is governed by optical factors other than CDOM absorption, e.g., absorption by suspended matter and chlorophyll and scattering by inorganic suspended material. Kratzer and Tett (2009) found that CDOM absorption is the dominant optical component in the Baltic Proper, but that inorganic suspended matter is responsible for most of the variability in light attenuation, especially in coastal waters. Furthermore, phytoplankton had a rather small influence on the attenuation of light due to the high background CDOM absorption. The phytoplankton biomass is higher in relation to the CDOM concentration in the BP than in the GB (unpublished results). Therefore, relatively seen, the phytoplankton biomass in the GB has less influence, and CDOM absorption has a higher influence on the Secchi depth than in the BP. Secchi depth is often used as an indicator for eutrophication, e.g., by HELCOM (2009), but this may be misleading as chlorophyll is not the only component contributing to changes in Secchi depth. We strongly recommend that CDOM absorption or CDOM fluorescence be included in coastal monitoring programs as a standard parameter, to increase the value of the concurrent Secchi depth measurements and to contribute to the development of improved remote sensing algorithms.

In GB, bacterial production is stimulated by river inflow of humic substances (Andersson et al. 2015). Hence, if climate change leads to a brownification of the Baltic Sea, bacterial production is likely to increase, whereas primary production may perhaps decrease, since phytoplankton need light, unlike most bacteria (Ask et al. 2009; Karlsson et al. 2009; Lefébure et al. 2013). If brownification is combined with decreased salinity in the future, conditions in the BP may come to resemble current environmental condition in the GB, except for the warming.

## Conclusion

Our empirical relationships show that there is a general decoupling between CDOM and DOC in the Baltic Sea, but that robust regional or local relationships can be found. We conclude that the CDOM pools in our study are mostly of terrestrial origin in both GB and BP, despite with different optical relationships, signals, and properties. Studies of CDOM effects on Secchi depth are especially important for the Baltic Sea in the contexts of climate change. The influence of different optical parameters on the Secchi depth, bacterial production, and primary production are central issues to study for a deeper knowledge of the likely effects of climate change, also in other seas with high terrestrial input of DOM. To validate 3-D ecological models used to project the future effects of climate change, the high spatial and temporal coverage of surface chlorophyll distribution in the Baltic Sea that only remote sensing can provide is needed. The development of remote sensing algorithms and the use of CDOM measurements within monitoring programs would be a step toward providing this for the GB.

## Electronic supplementary material

Supplementary material 1 (PDF 596 kb)
