# Supplementary material for: Relationships between colored dissolved organic matter and dissolved organic carbon in different coastal gradients of the Baltic Sea
Source: Ambio. 2015 May 28;44(Suppl 3):392–401. doi: 10.1007/s13280-015-0658-4 (PMC4447701; doi:10.1007/s13280-015-0658-4)
Supplement: Supplementary file 1 — Supplementary material 1 (PDF 596 kb) [file 13280_2015_658_MOESM1_ESM.pdf]

Electronic Supplementary Material

AMBIO

This supplementary material has not been copy edited by the publisher or the editorial office.

Title: **Relationships between coloured dissolved organic matter and dissolved organic carbon in different coastal gradients of the Baltic Sea**

Authors: E. Therese Harvey, Susanne Kratzer and Agneta Andersson

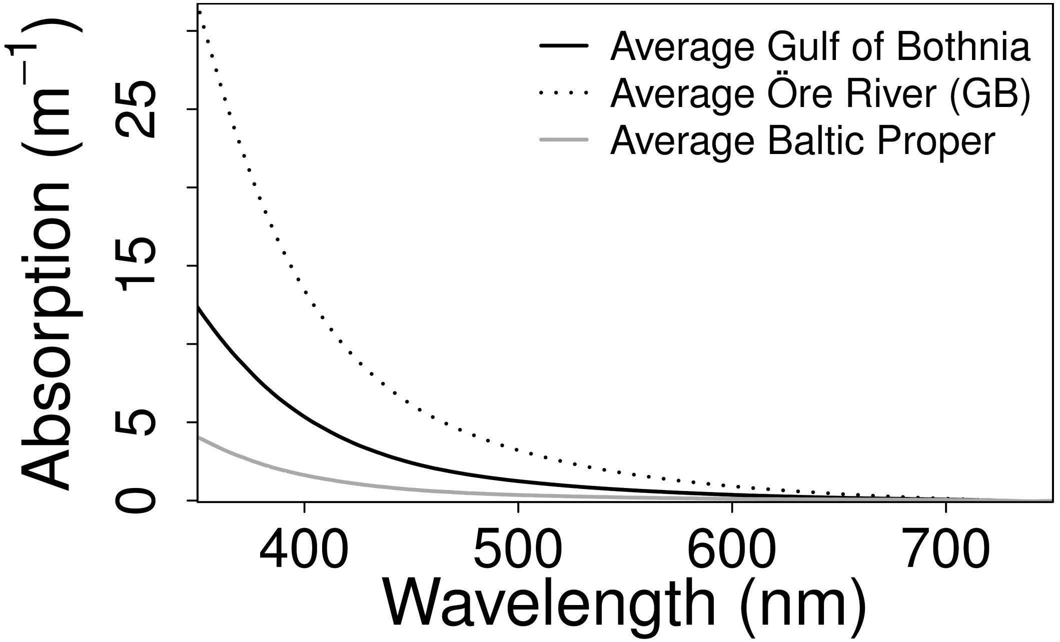

Fig. S1 Average CDOM absorption spectra for the Öre estuary in the Gulf of Bothnia (GB) (black line), the Öre River (black dashed line) and the Baltic Proper without the inner locations of Nyköping gradient (grey line).

Table S1 Descriptive data for CDOM absorption, g440, (m^{-1}), CDOM slope coefficient, *S* (m^{-1}), DOC (mg l^{-1}), Salinity, Secchi depth (m) and CDOM:DOC ratio (l m^{-1} mg^{-1}) for the studied gradients. Spring and summer data were available from the Öre estuary and the Himmerfjärden Bay. The first rows show the range, the second rows (bold) show the mean and its standard error (SEM) and the third rows give the standard deviation (in brackets) for each gradient

Area	Gradient/Estuary	Season		g440 (m^{-1})	*S* (m^{-1})	DOC (mg l^{-1})	Salinity	Secchi depth (m)	CDOM:DOC ratio (l m^{-1} mg^{-1})
**Gulf of Bothnia**	**Öre Estuary**	*Spring*	min-max	1.51 - 8.83	0.015 - 0.016	4.4 - 10.2	0.1 - 2.5	0.45 - 3.5	0.33 - 0.94
			mean ± SEM	**5.16 ± 0.47**	**0.015 ± 6e^{-5}**	**7.6 ± 0.51**	**1.5 ± 0.16**	**1.3 ± 0.18**	**0.64 ± 0.035**
			Stdev	(2.69)	(3.5e^{-4})	(2.09)	(0.01)	(0.96)	(0.199)
		Summer	min-max	0.75 - 7.58	0.015 - 0.017	3.8 - 9.9	0.2 - 2.9	1.1 - 6.0	0.19-0.84
			mean ± SEM	**1.93 ± 0.13**	**0.016 ± 4e^{-5}**	**4.9 ± 0.12**	**2.4 ± 0.05**	**3.6 ± 0.08**	**0.35 ± 0.013**
			Stdev	(1.60)	(5.5e^{-4})	(1.38)	(0.52)	(0.89)	(0.157)
**Baltic Proper**	**Östhammar gradient**	*Summer*	min-max	0.48 - 1.24	0.015 - 0.018	4.1 - 6.8	4.2 -5.3	0.9 - 12.8	0.14-0.17
			mean ± SEM	**0.80 ± 0.04**	**0.017 ± 1.6e^{-4}**	**5.2 ± 0.36**	**4.8 ± 0.06**	**4 .0 ± 0.66**	**0.15 ± 0.005**
			Stdev	(0.20)	(7.2e^{-4})	(0.88)	(0.28)	(3.04)	(0.013)
	**Himmerfjärden bay**	*Spring*	min-max	0.33 - 0.60	0.016 - 0.020	4.1 - 5.0	5.7 - 6.3	3.5 - 9.0	0.08-0.13
			mean ± SEM	**0.44 ± 0.02**	**0.018 ± 1.7e^{-4}**	**4.5 ± 0.13**	**6.1 ± 0.04**	**6.9 ± 0.25**	**0.10 ± 0.005**
			Stdev	(0.08)	(9.3e^{-4})	(0.33)	(0.19)	(1.36)	(0.018)
		Summer	min-max	0.34 - 1.57	0.013 - 0.018	4.3 - 5.4	3.7 - 6.3	1.7 - 5.3	0.08-0.16
			mean ± SEM	**0.63 ± 0.04**	**0.016 ± 1.6e^{-4}**	**4.7 ± 0.10**	**5.6 ± 0.11**	**3.5 ± 0.18**	**0.12 ± 0.005**
			Stdev	(0.24)	(0.0011)	(0.40)	(0.60)	(0.98)	(0.021)
	**Nyköping gradient**	*Summer*	min-max	0.45 - 4.12	0.016 - 0.018	4.3 - 12.8	0.3 - 6.1	0.7 - 5.9	0.10-0.32
			mean ± SEM	**1.55 ± 0.22**	**0.017 ± 1.5e^{-4}**	**7.5 ± 0.87**	**2.3 ± 0.51**	**2.3 ± 0.37**	**0.17 ± 0.018**
			Stdev	(1.00)	(6.6e^{-4})	(3.12)	(2.29)	(1.67)	(0.066)
	**Norrköping area**	*Summer*	min-max	0.36 - 1.95	0.013 - 0.021	4.5 - 7.4	3.0 - 6.5	1.0 - 6.8	0.08-0.26
			mean ± SEM	**0.80 ± 0.06**	**0.018 ± 2.4e^{-4}**	**5.4 ± 0.75**	**5.3 ± 0.0.17**	**2.8 ± 0.21**	**0.15 ± 0.012**
			Stdev	(0.38)	(0.0015)	(0.18)	(1.04)	(1.29)	(0.050)
